# Structuring of specialized treatment applied to people with suicidea ttemptin an academic psychiatric service in Rio de Janeiro city

**DOI:** 10.1192/j.eurpsy.2022.2183

**Published:** 2022-09-01

**Authors:** J. Jaber

**Affiliations:** Clínica Jorge Jaber, Saúde Mental, Rio de Janeiro, Brazil

## Abstract

**Introduction:**

Description of a specialized treatment program for people attempting suicide in an academic health service focused on psychiatry and drug addiction in the city of Rio de Janeiro.

**Objectives:**

Describe actions developed to treat suicidal behavior.

**Methods:**

Based on a survey of the prevalence of suicidal behavior in the Brazilian population over a lifetime, reaching a total of 12,000 cases per year of the Brazilian population, this academic service of psychiatry and drug addiction established the following actions for hospitalized patients: 24-hour surveillance, reduced access to methods of committing suicide (forks and knives removal, shoelaces and ropes removal), strengthening the GVV (Life Valuation Group), strengthening the Cognitive Behavior Therapy application groups, conducting group dynamics, lectures, art therapy and physical activities.

**Results:**

Of 370 patients admitted to this service from January 1^st^, 2019 to September 1^st^, 2019, 137 had suicidal behavior and only 2 died.

**Conclusions:**

Of these two cases, one abandoned treatment and the other occurred during the treatment period.

**Disclosure:**

No significant relationships.

